# Consume, Contribute, and Create: Succeeding as a Learner and Educator in the Digital Era

**DOI:** 10.14797/mdcvj.1083

**Published:** 2022-06-03

**Authors:** Gurleen Kaur, Daniel Ambinder, Amit Goyal

**Affiliations:** 1 Brigham and Women’s Hospital, Boston, Massachusetts, US; 2 Johns Hopkins Hospital, Baltimore, Maryland, US; 3 Cleveland Clinic, Cleveland, Ohio, US

**Keywords:** medical education, adult learning theory, podcasts, social media, tweetorials

## Abstract

From medical student to professorship, the practice of medicine requires lifelong learning. The unforgivingly rapid expansion of medical literature often renders traditional educational resources quickly outdated if not altogether obsolete. Conversely, increasingly popular digital platforms are easily accessible and quickly updated, offering vital adjuncts to traditional resources for the modern student. Further, platforms such as podcasts and social media may be particularly well suited for adult learners who tend to be problem centered, self-directed, internally motivated, and time constrained. Social media empowers all participants, thereby blurring the boundaries between learners and educators. Here we review novel digital educational platforms, discussing both potential benefits and pitfalls, and then provide a three-pillared approach—consume, contribute, and create—to help the modern medical professional harness the potential of both traditional and novel resources to succeed as both a learner and educator.

## Introduction

“To study the phenomenon of disease without books is to sail an uncharted sea, while to study books without patients is not to go to sea at all.” – Sir William Osler

While bedside teaching remains irreplaceable, the advent and propagation of digital medical education have opened new avenues for the dissemination of science and knowledge. From early trainees to seasoned professors, the practice of medicine requires lifelong learning.

**Figure 1:**
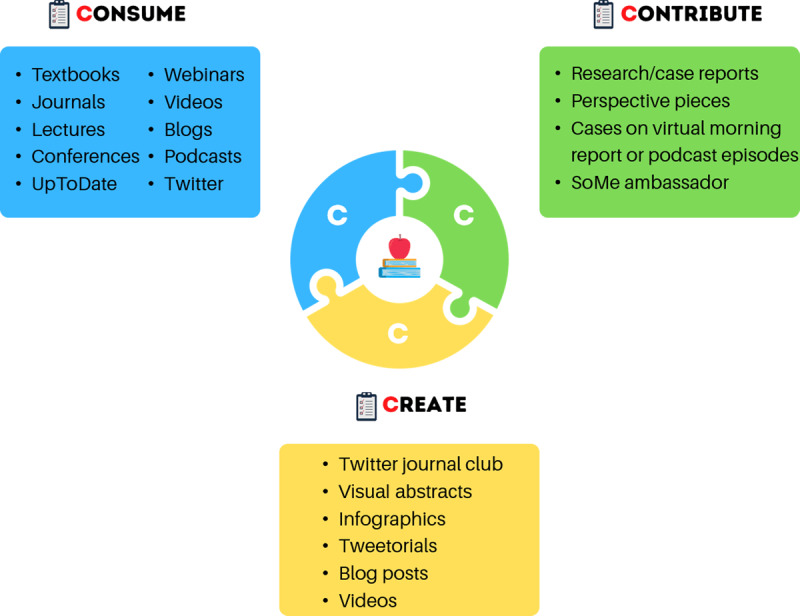
The three C’s—consume, contribute, and create—for succeeding as a learner and educator. SoMe: social media

Adult learners tend to be problem centered, self-directed, internally motivated, and time constrained and so should engage with the plethora of available resources in ways that best fit their unique learning styles, goals, and schedules.^1^ In this review, we provide a three-pillared approach—consume, contribute, and create—for navigating modern post-graduate medical education and succeeding as both a learner and an educator (Figure 1).

## Consume

Medical trainees are inundated with a variety of resources from which to learn and expand their knowledge. Traditional educational resources, including textbooks, journals, lectures, and conferences, serve an important role in forming core knowledge and incorporating evidence-based medicine into routine clinical practice. However, with the rapid growth of scientific literature, traditional means like textbooks quickly become outdated. The last decade has seen an explosion in digital education resources such as podcasts, videos, blogs, and teaching via social media platforms such as Twitter and Instagram.^2^ The popularization of free and open access medical education has revolutionized our ability to learn through several interactive formats featuring rapidly updated information, allowing users to keep up with the pace of scientific discovery. Access to expert opinion is now readily available through multiple modalities. Further, users can garner expert opinion while also engaging in discussions within the scientific community at large. These new digital educational modalities also have been referred to as organic digital education, a term coined by Rodman and Trivedi, which refers to the transition from traditionally hierarchical medical education to a democratized form of learning.^3^

### Podcasts

Medical podcasts have become an increasingly popular form of education because they are easily accessible and particularly well suited for time-constrained adult learners.^3^ Medical education podcasts are consumed by a broad audience in terms of both stage of career and geographical location. Data from The Curbsiders, an internal medicine podcast, identified their audience as 15% students, 23% residents or fellows, 20% advanced practitioners, and 38% faculty or post-training physicians.^4^ Furthermore, compiled data from three major internal medicine podcasts—Bedside Rounds, CORE IM, and The Curbsiders—showed that episodes were listened to in 192 of 297 countries worldwide.^5^

Podcasts are an efficient and convenient way to gather both fundamental concepts and nuanced knowledge about broad content areas through engaging discourse. Such platforms allow for content to be more easily consumed compared to modes of traditional medical education. The benefits of podcasts have been described in two qualitative studies exploring how and why listeners engage with medical podcasts.^6,7^ While the dialogues in podcasts are unidirectional, the experience of listening has still been described as a social phenomenon, allowing for connection with the larger professional community.^6,7^ Podcast hosts and invited speakers can serve as role models for both clinical practice and demonstrations of the hidden curriculum of professionalism and mutual respect.

As the medical community increasingly adopts podcasts for learning, evaluating their effectiveness as an educational tool becomes increasingly important. In a randomized controlled trial of students assigned to podcasts versus text-based learning, podcast users had a higher gain of knowledge and greater satisfaction with podcasts.^8^ Similarly, when CardioNerds, a cardiovascular digital education platform, surveyed its podcast newsletter subscribers about the impact of medical podcasts, 95% indicated that medical podcasts have added to their knowledge base, 93% indicated that medical podcasts have made them better educators, and 89% indicated that medical podcasts have changed their clinical practice (unpublished data). This is consistent when specifically assessing attitudes towards podcasts in a narrower subset of learners. For example, 75% of cardiology fellows who participated in a cardiology case-based podcast series, the CardioNerds Case Reports (CNCR), either strongly agreed or agreed to the statement that listening to medical podcasts has changed their clinical practice. Of these fellows, 94% reported that medical podcasts added to their knowledge base.^9^

**Figure 2:**
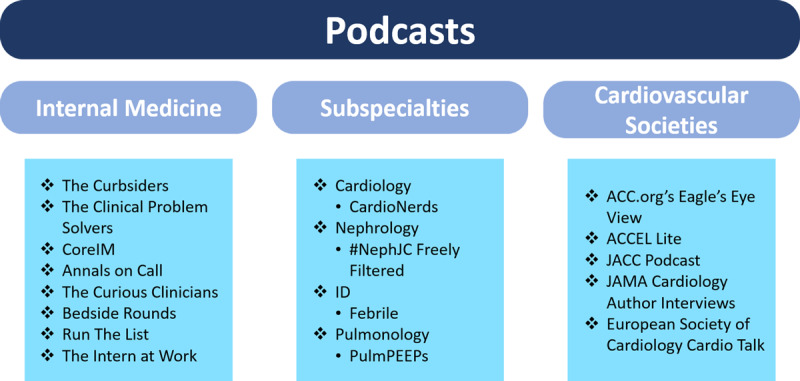
Examples of medical education podcasts related to internal medicine, subspecialties, and cardiovascular societies. ACC: American College of Cardiology; JACC: Journal of the American College of Cardiology; JAMA: Journal of the American Medical Association

The breadth and design of medical podcasts are widely variable, from clinical reasoning discussions to interviews with content experts to discussions of primary scientific literature. They also are leveraged by professional societies as a supplement to their journals (Figure 2). The variety of available podcasts allows listeners to choose episodes that meet their individualized learning needs and goals. Furthermore, the flexibility of the format allows for engagement based on the listeners’ schedules. As such, podcasts are particularly well suited for adult learners who are typically self-directed and time constrained.

### Social Media

Social media platforms such as Twitter are another way for a global audience of medical professionals to stay abreast of ongoing scientific advances. Scientific journals have established a concerted presence on Twitter, often sharing articles on social media before print journals reach readers. Further, dissemination of research on social media may lead to greater article downloads and citations.^10,11,12^ Twitter conversations allow for democratization of information from the latest scientific meetings; these live threads along with Twitter journal clubs, hosted by different organizations and professional societies, give direct access to content experts in the field. The impact of Twitter content can be measured via impressions (number of times a tweet appears to users) and engagements (number of times a user interacted with the tweet), and the practice of attaching hashtags (#) to words allows for easily searchable content. Specifically, “#Cardiotwitter” was used in 53,500 tweets and accumulated 162.9 million impressions from October 2017 to September 2018.^13^

Social media has created a space for free-form discourse, empowering everyone to share ideas and knowledge. These tectonic shifts in how the medical community engages with one another have blurred the boundaries between learners and educators. Tweetorials—composed of a series of threaded tweets on Twitter—are another form of organic digital education that have democratized medical education by shifting away from the traditionally hierarchical model of education. Tweetorials disseminate content about a specific topic and often incorporate several aspects of multimedia, such as links to videos and articles as well as polls, to allow for active learning and the opportunity for discussion, questions, and feedback.^14^ In this mode of asynchronous education, learners have the liberty to either consume the information, refer back to the material on their own time, or engage in the discussion via comments on the thread, allowing for spaced learning. In a study evaluating residents’ attitudes towards Twitter feeds, 69% agreed that Twitter enhanced their overall education in residency.^15^

Given the vast resources available for medical education, sorting through content and deciding which sources to use can be a daunting experience. Furthermore, learners must critically decipher the quality and veracity of all content and their sources. The realm of digital education has further expanded during the COVID-19 pandemic, with an increase in the number of webinars and online meetings, leading to various modes for both synchronous and asynchronous engagement. This has led to the challenge of balancing the desire to learn from these myriad platforms with time for self-care. As adult learners, we must carefully select from the overabundance of educational resources according to how we learn best as individuals and balance it with personal well-being.

## Contribute

Becoming an active consumer of medical education can be a fulfilling experience that leads to professional growth, but actually contributing to traditional or novel educational platforms can generate more active engagement, greater content mastery, and enhanced professional development. Traditional ways to contribute include publishing original research or case reports in peer-review journals, thus contributing to scientific literature and disseminating information to a targeted audience. Contribution within traditional constructs can also be in the form of advocacy. For example, the *Journal of the American College of Cardiology* features an early career section with perspective pieces. The American College of Cardiology (ACC) also features a Fellows in Training (FIT) newsletter with articles on the online webpage spanning different topics, including an interview series called “Conversations with Cardiologists.”

The increasing popularity of digital education platforms enables dissemination of science, education, and advocacy to a global audience spanning all levels of training and practice within all variety of disciplines. The Clinical Problem Solvers—a podcast and educational community focused on diagnostic reasoning—designed a virtual, multi-institutional, case-based diagnostic reasoning platform in 2020 known as Virtual Morning Report (VMR).^16^ These sessions allow healthcare professionals at any level of training to contribute to live case discussions alongside faculty facilitators. Contributing to platforms such as VMR allows access to experts outside of one’s own institutions, provides positive role models within an inclusive learning environment, and creates a sense of community. Such initiatives are generalizable to all specialties within medicine; for example, one weekly session of VMR is dedicated to discussion of neurology cases.^17^

Another way to contribute to digital medical education is by sharing cases on podcasts such as the CardioNerds. The CNCR series allows trainees to submit educational cases that are then featured on the podcast in an engaging format. Of the cardiology fellows contributing to the CNCR series, 89.6% indicated that it was an effective way to build a sense of community during the COVID-19 pandemic and 92% that it was effective in teaching cardiovascular concepts.^9^ Another way to contribute to case-report discussions is through social media platforms.^18^ Threads on social media often discuss cases with links to references and important papers in the field. However, as with any open-access format, it is necessary to be wary of quality checks, ensure that patient privacy is protected in accordance with the Health Insurance Portability and Accountability Act (HIPPA), adhere to institutional guidelines, and use established best practices.^13^

A key benefit of digital communities is the ability to promote diversity, equity, and inclusion both deliberately and as part of an important hidden curriculum. The digital space breaks the traditional barriers of our individual institutions, allowing for increased visibility to individuals across all backgrounds. The Clinical Problem Solvers VMR, described above, was soon expanded to include a Global VMR designed specifically for medical trainees outside the United States, where international team members provide translations and participants are encouraged to share thoughts in the language of their choosing. In addition, the CardioNerds Narratives in Cardiology series, a collaboration with the Pennsylvania ACC Chapter and FIT section, features fellows in conversation with leading figures in cardiology to deliberately promote diversity, equity, and inclusion.^19^ Podcasts can be designed intentionally to give listeners access to diverse perspectives, promote reflection, and normalize conversations about racial justice.^20^

Another way to contribute is to join discussions hosted by professional societies on social media platforms. In July 2018, the ACC FIT council organized tweetorials through the hashtag #FITSurvivalGuide on several fundamental clinical topics.^13^ Trainees also can serve as social media ambassadors for professional societies during annual conferences. Serving in this role involves providing live coverage of the meeting and setting up the stage for discussions related to late-breaking clinical trials, allowing for real-time discourse about emerging research. Individuals who may not have come across the studies are able to read summaries through social media and can actively participate in the conversation, allowing for critical appraisal of the literature and post-publication peer review.^21^ Contributing not only enhances one’s knowledge but also enables networking and professional development, creating a global community of learners and educators.

## Create

Conventional resources used for the past several decades typically involve unilateral top-down learning with clear distinction between the learner and the educator. One advantage of free, open access, and digital medical education methods is the capacity for bidirectional flow of education, with opportunities both for trainees to disseminate quality education and for practicing faculty to engage in lifelong learning. This collaborative and empowering environment allows learners to create novel content in the digital space.

Creating and assessing digital content is often not specifically addressed within traditional training programs, and many may lack formal guidance. Creation of digital education communities has prompted trainees to come together to gain skills in this realm. The Nephrology Social Media Collective (NSMC) created a 1-year internship in 2015 that offers mentorship for the creation of digital education products (DEPs), such as visual abstracts, infographics, blog posts, and Twitter-based journal clubs.^22^ Likewise, the CardioNerds Academy established in 2020 allows students, residents, and fellows to gain skills in creating DEPs such as tweetorials, infographics, and online journal clubs with the goal of combining content creation with personal and professional development. While NSMC and CardioNerds are examples of two independent virtual educational communities, the Innovations in Media and Educational Delivery Initiative at Beth Israel Deaconess Medical Center in Boston, Massachusetts, is an example of a formal digital educational skills curriculum within a traditional internal medicine training program.^23^

**Figure 3:**
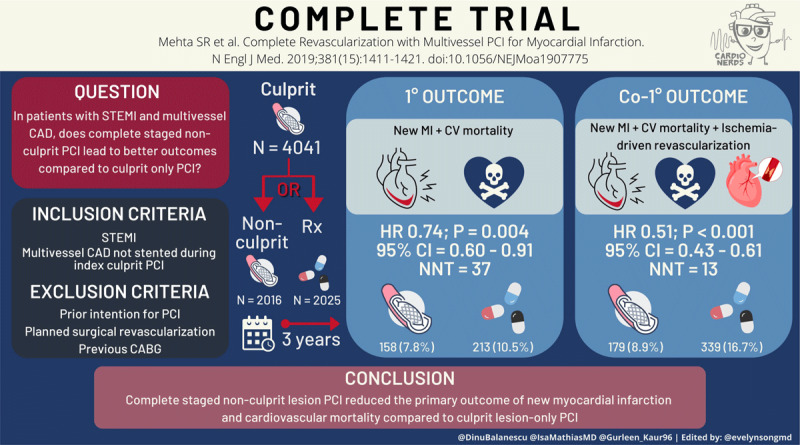
Visual abstract for CardioNerds’ Twitter journal club, #CardsJC. Reprinted with permission from CardioNerds (www.cardionerds.com). STEMI: ST-elevated myocardial infarction; CAD: coronary artery disease; PCI: percutaneous coronary intervention; CABG: coronary artery bypass graph; MI: myocardial infarction; CV: cardiovascular

Both the NSMC and CardioNerds Academy have established regular journal clubs, hosted on Twitter, in which the audience can follow the discussion using the hashtags #NephJC and #CardsJC, respectively.^24,25^ Twitter journal clubs cultivate access to clinical trial leadership, content experts, and perspectives from a diverse audience, and trainees can create content and design tweets with questions to elevate the scientific discourse around pivotal clinical trials. For example, NSMC members (for #NephJC) and CardioNerds Academy members (for #CardsJC) create a trial summary, infographic (Figure 3), and Twitter thread for each journal club, and the resulting content is available both synchronously during the event and asynchronously for later consumption.

**Figure 4:**
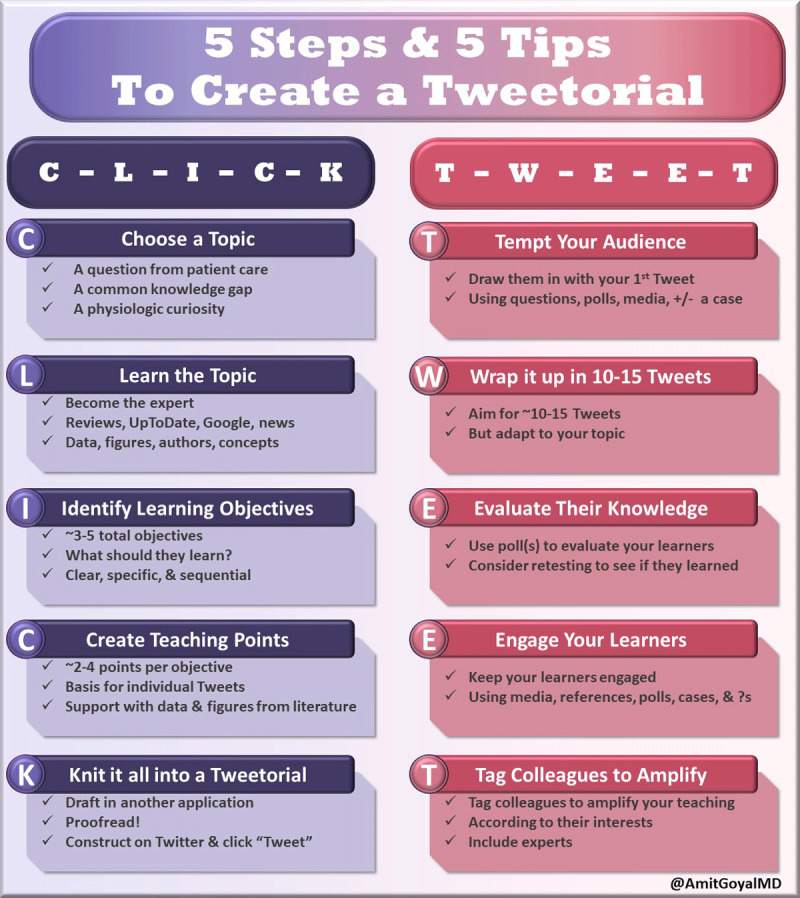
Infographic on 5 Steps and 5 Tips to create a tweetorial. Reprinted with permission from CardioNerds (www.cardionerds.com).

Tweetorials, described earlier, are another method of content creation that allow students, residents, and fellows to teach thousands of people and reach audiences different from the typical learning classroom. One way learners can create their own tweetorials is through the proposed “CLICK TWEET” method (Figure 4).^26^ It is also important to assess the impact of posted journal clubs and tweetorials just as one would solicit feedback after giving a “chalk talk,” lecture, or grand rounds. The dissemination on social media also lends itself to organic peer review from both colleagues and content experts. Content creation requires an iterative process of learning from experience and evolving over time with practice and feedback, all leading to greater content mastery.

The experience and knowledge gained through content creation can be applied directly to the clinical environment both for patient care and teaching learners on hospital wards. Infographics and tweetorials can be used on rounds and facilitate the development of “chalk talks” on various topics.

## Caveats of Consuming, Contributing, and Creating in the Digital Era

The use of social media in education and the rise of digital resources does have some important limitations to consider. When consuming content in the digital space, one must be cognizant about the possibility of misinformation and lack of validity because digital content may not have undergone the same rigor of peer review as traditional resources. Reassuringly, the open format of digital media allows for fact checking from learners, educators, and often content experts, with the resulting discourse serving as an organic peer review.

These limitations also apply when contributing and creating content. Given the wide-reaching influence of publicly available content, establishing accuracy is fundamental as a creator and educator. Ways to ensure dissemination of the highest quality material include giving credit to original creators, listing relevant references, and “tagging” content experts. Collaborating with peers or experts can enable a review process to confirm validity of the content that is created and avoid the spread of misinformation.^13^ Creation of new content that is disseminated widely across online platforms requires great responsibility, specifically with regard to protecting patient privacy, adhering to HIPAA obligations, and complying with institutional guidelines.

Another limitation to learning in the digital era is the surplus of content that exists. Given the abundance of resources in both digital and traditional forms, it can be challenging to navigate among options. As educational content becomes more easily accessible, educators and students alike must learn how to best integrate the plethora of information with day-to-day activities.

## Conclusion

The evolution of medical education has blurred the distinction between learner and educator, and therefore the steps to success as either one are inherently intertwined. The three-pillared approach of consuming, contributing, and creating does not represent three isolated ways to engage but, rather, a continuum of growth. While traditional methods of medical education and bedside instruction remain irreplaceable, the contemporary era of digital media opens a plethora of opportunities to chart a route through Osler’s sea of patient care and meet the unique needs of adult learners. While the digital space may still be foreign to some, we encourage learners and educators to explore the possibilities and harness the opportunities by consuming, contributing, and creating.

## Key Points

- Succeeding as a modern learner and educator requires a personalized approach using both traditional and novel digital resources.
- Learners and educators can harness the potential of available educational resources by consuming, contributing, and creating.
- As adult learners, medical professionals are problem-centered, self-directed, internally motivated, and time-constrained and so should engage with the plethora of available resources in ways that best fit their unique learning styles, goals, and schedules.
- Digital educational platforms empower all participants, thereby blurring the boundaries between learners and educators.
- The open-source format of digital platforms warrants special consideration for ensuring content validity, combating misinformation, protecting patient privacy, and upholding the utmost professionalism.
